# Human Rhinovirus Group C in Hospitalized Children, Singapore

**DOI:** 10.3201/eid1508.090321

**Published:** 2009-08

**Authors:** Boon-Huan Tan, Liat-Hui Loo, Elizabeth Ai-Sim Lim, Shirley Lay-Kheng Seah, Raymond T.P. Lin, Nancy W.S. Tee, Richard J. Sugrue

**Affiliations:** DSO National Laboratories, Singapore (B.-H. Tan, E.A.-S. Lim, S.L.-K. Seah); Nanyang Technological University, Singapore (L.-H. Loo, R.J. Sugrue); Kandang Kerbau Women’s and Children’s Hospital, Singapore (L.-H. Loo, N.W.S. Tee); National University Hospital, Singapore (R.T.P. Lin)

**Keywords:** Rhinovirus, rhinovirus group C, pediatric patients, children, Singapore, viruses, letter

**To the Editor:** Human rhinovirus (HRV) is a common etiologic agent of upper respiratory tract infections and is associated with symptoms such as asthma and wheezing. HRV has >100 serotypes, and recently, several groups reported a new HRV group C (HRV-C) in children that is associated with more severe respiratory infections (*1**–**5*). We examined the incidence of respiratory viruses in children hospitalized in Kandang Kerbau Women’s and Children’s Hospital, Singapore (*6**,**7*). These studies also identified human metapneumovirus and human bocavirus (HBoV) among children in Singapore. We recently performed a retrospective study by using PCR-based testing (*8*) to identify HRV, in particular HRV-C, in these patients. From October 2005 through March 2007, a total of 500 nasopharyngeal swab specimens from pediatric patients (age range 1 month through 12 years) were collected and tested for HRVs.

PCR-based testing identified HRV with an incidence rate of 12.8% (64/500), the highest incidence rate in Singapore, compared with incidence rates of other respiratory viruses reported in the same study (*7*). Of the HRV-positive patients, 31 (48.4%) of 64 had symptoms of lower respiratory tract infections (LRTIs) and 16 (25%) of 64 had symptoms of upper respiratory tract infections. Ten patients infected with HRV were co-infected with a second respiratory virus, HBoV (8/10) or respiratory syncytial virus (RSV) (2/10).

HRV-C was detected by molecular serotyping as described (*3*). Briefly, the first PCR was performed with the forward primer P1–1 (5′-CAA GCA CTT CTG TYW CCC C-3′) and the reverse primer P3–1 (5′-ACG GAC ACC CAA AGT AG-3′). A second heminested PCR was performed with forward primer P1–1 but with 3 different reverse primers, P2–1 (5′-TTA GCC ACA TTC AGG GGC-3′), P2–2 (5′-TTA GCC ACA TTC AGG AGC C-3′), and P2–3 (5′-TTA GCC GCA TTC AGG GG-3′). PCR amplicons were sequenced by using the P1–1 primer. DNA sequences were blasted by using the National Center for Biotechnology Information database (Bethesda, MD, USA) and aligned with available sequences by using Clustal X version 1.83 software (www.bips.u-strasbg.fr/fr/documentation/clustalx). All protocols are available on request.

A phylogenetic tree (GenBank accession nos. FJ645828–FJ645771) was constructed by using neighbor-joining method with 1,000 bootstrap replicates and MEGA version 4 software (*9*). The tree showed similar branching of known HRVs into serogroups (HRV-A, HRV-B, and HRV-C) as described (*3*). Forty-seven (73%) of the 64 HRV specimens from Singapore were grouped into HRV-A, 9 (14%) into HRV-B, and 2 (3%) into HRV-C. We also found a cluster of 10 HRV-A strains (Figure) diverging from the reference HRV-A strains. This finding suggests that these strains could be new strains of the HRV-A, as reported (*3*). We could not determine virus subtype for 6 specimens, possibly because of low virus load.

**Figure:**
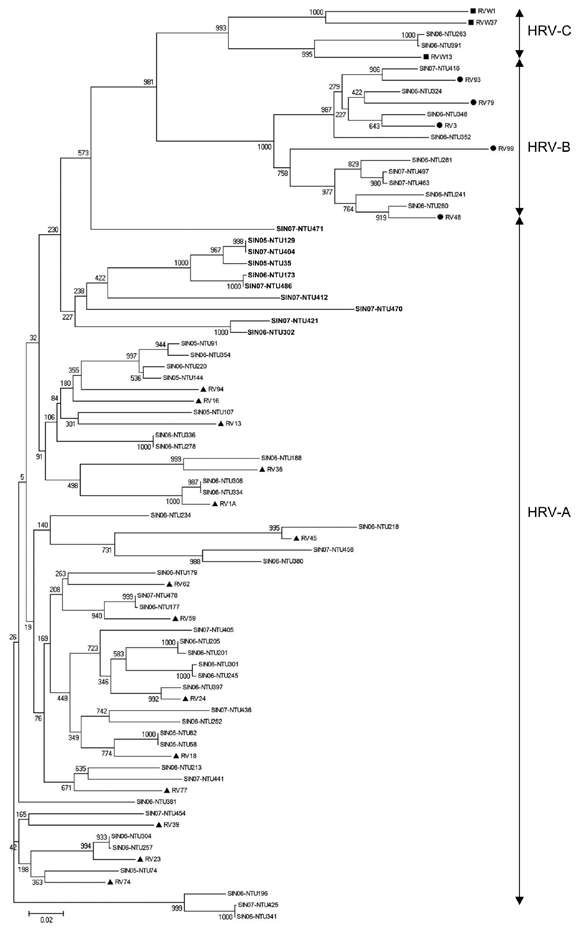
Phylogenetic analysis of human rhinoviruses (HRVs) from Singapore based on nucleotide sequences of the 5′ noncoding region. The tree was constructed by using the neighbor-joining method with 1,000 bootstrapped replicates generated by MEGA version 4 software (*9*). Sequences (GenBank accession nos. FJ645828–FJ645771) of viruses from Singapore (SIN) are indicated, where the 2 numbers represent the year the specimen was collected, and NTU (Nanyang Technological University) followed by 3 numbers represents the specimen number. Representative strains of HRV-C are indicated by squares, HRV-B by circles, and HRV-A by triangles. RV indicates rhinovirus strains, followed by the serotype no. These sequences were obtained from the report by Lee et al. (*3*). **Boldface** indicates a cluster of 10 HRV-A strains that diverged from reference HRV-A strains. Scale bar indicates nucleotide substitutions per site.

Our results confirm that HRV infections in Singapore are caused mainly by HRV-A. An increase in HRV-C infections with the onset of winter has been reported in the People’s Republic of China (26%) (*5*) and the Hong Kong Special Administrative Region of China (80%) (*2*). These findings indicate that the incidence of HRV-C infections is seasonal, which may account for the apparent low rates of HRV infection in Singapore. However, the incidence rate for HRV-C infections in Singapore was higher than that for HRV-C infections in Australia (1.4%) (*4*), which has a clearly defined winter season.

The 2 patients in which HRV-C was detected had asthma (virus strain SING-06–263) and bronchiolitis (virus strain SING-06–291). These observations are consistent with reports of HRV-C in patients with severe wheezing (*2**,**4**,**10*). We also detected co-infection with another virus in 10 patients infected with HRV. Of these 10 co-infections, HRV-A was detected in 7 patients; 5 were co-infected with HBoV (2 patients had LRTIs, 2 had upper respiratory tract infections, and 1 had undefined symptoms), and 2 were co-infected with RSV (both patients had symptoms of LRTIs). Of the other 3 patients co-infected with HRV and HBoV, 1 was infected with HRV-B (had LRTI), 1 with HRV-C (had LRTI), and 1 with an untypeable HRV (had undefined symptoms). Co-infections of HRV with RSV (*4**,**5*) and HBoV (*4*) have been reported.

Although the clinical role of these co-infections needs to be clarified, these studies suggest that co-infections may result in more severe disease symptoms. The role of HRV-C in causing illness among the children of Singapore will require further study.
